# PD‐1 expression on the surface of peripheral blood CD4^+^ T cell and its association with the prognosis of patients with diffuse large B‐cell lymphoma

**DOI:** 10.1002/cam4.874

**Published:** 2016-10-05

**Authors:** Wei Zhang, Jie‐Fei Bai, Meng‐Xuan Zuo, Xin‐Xin Cao, Miao Chen, Yan Zhang, Xiao Han, Ding‐Rong Zhong, Dao‐Bin Zhou

**Affiliations:** ^1^ Department of HematologyPeking Union Medical College HospitalChinese Academy of Medical Sciences and Peking Union Medical CollegeBeijingChina; ^2^ Department of PathologyPeking Union Medical College HospitalChinese Academy of Medical Sciences and Peking Union Medical CollegeBeijingChina

**Keywords:** Diffuse large B‐cell lymphoma, programmed death‐1, T cells, tumor microenvironment

## Abstract

The aim of the study was to investigate the relationship between PD‐1 expression on the surface of CD4^+^ T cells and prognosis of patients with diffuse large B‐cell lymphoma (DLBCL). Sixty patients who were newly diagnosed with DLBCL and 39 healthy controls were enrolled. In CD4^+^ T cells of DLBCL patients, the median MFI of PD‐1 were 541.5 (range: 348.25–758.75), significantly higher than 250 (range: 211–326) in healthy controls (*P* < 0.001). The ZAP70, PI3K, and NFAT mRNA expression levels of patients were 0.47, 0.47, and 0.62 times, respectively, of those of the healthy controls (*P* < 0.05). In patients with the percentage of PD‐1 on CD4^+^ T cells ≥30.25%, their EFS and OS were significantly lower than patients with PD‐1^+^ CD4^+^ T cells <30.25% (*P* < 0.05). The possible explanation is that high PD‐1 expression on CD4^+^ cells of DLBCL patients may impair T‐cell function and thus contribute to poor prognosis. There was no relationship between PD‐1 surface expression on CD4^+^ T cells and PD‐1 expression within the biopsy of tumor microenvironments from DLBCL patients.

## Introduction

Diffuse large B‐cell lymphoma (DLBCL) is the most common form of non‐Hodgkin's lymphoma, accounting for 36.2% of all cases in China 1. Currently, the most widely used therapy is R‐CHOP (rituximab + cyclophosphamide + doxorubicin + vindesine + prednisone) and although 50–60% of DLBCL patients achieve complete remission, 30–40% of patients still relapse (~10% of patients with refractory) 2. Exploration of DLBCL pathogenesis and discovery of appropriate drug targets could improve response rates and the survival time of patients with DLBCL. PD‐1 (CD279) is an immunoglobulin superfamily type I transmembrane glycoprotein, which contains an N‐terminal extracellular IgV‐like region, a transmembrane region, and a cytoplasmic region. PD‐1 expression is very low in resting cells, but after cell activation, PD‐1 is widely expressed on T lymphocytes, B lymphocytes, NK cells, NK/T cells, and macrophages. PD‐1 is an inhibitory molecule of the B7 family and PD‐1 overexpression can cause inactivation of T cells via phosphatase SHP1 and SHP2 recruitment, which then inhibits the zeta chain accessory protein kinase (ZAP70)/nuclear factor of activated T (NFAT) pathway 3. PD‐1 also directly inhibits phosphatidylinositol 3‐kinase (PI3K) and the corresponding downstream Akt activity, thereby inhibiting T lymphocyte proliferation 4. Previous studies have found that PD‐1 expression is significantly increased in peripheral blood CD3^+^ T lymphocytes of patients with Hodgkin's lymphoma, chronic lymphocytic leukemia, and multiple myeloma. Immunomodulatory therapies, such as lenalidomide or panobinostat, significantly reduced PD‐1 surface expression on T lymphocytes of patients 5, 6, 7. Armand P discovered that PD‐1 inhibitor administration was linked to a total remission rate of 51% in 35 DLBCL patients with residual lesions after autologous hematopoietic stem cell transplantation 8. These findings suggest that DLBCL patients may also have abnormal expression of PD‐1 on peripheral blood T lymphocytes. Although PD‐1 was expressed on the surfaces of both CD4^+^ and CD8^+^ T cells, higher levels of PD‐1 expression on the surface of CD4^+^ T cells have been noted 9, which is consistent with CD4^+^ T cells having an important role in immune function. In this study, we explored the relationship between PD‐1 expression on peripheral blood CD4^+^ T cells and the prognosis of DLBCL patients.

## Materials and Methods

### Patients

From July 2014 to June 2015, the pathologies of 60 untreated DLBCL patients were confirmed by pathologist of Peking Union Medical College Hospital (PUMCH) and were excluded based on chronic hepatitis B, cytomegalovirus (CMV), and Epstein–Barr virus (EBV) infection. Thirty‐nine healthy control subjects were randomly selected from medical examination center of PUMCH. All participants signed informed consent forms. Peripheral blood samples were collected from patients and healthy controls. The pathological specimens of patients include lymph nodes or also extranodal sites were fixed in formalin and embedded in paraffin. The study was approved by the Ethics Committee of PUMCH.

### Flow cytometry

About 1 × 10^6^ peripheral blood mononuclear cells were collected and incubated with staining antibodies for 25 min, washed twice in 2 mL PBS cells (300 g, 5 min), and finally fixed with a 1% formalin. CD3 (Clone SP34‐2; PerCP), CD4 (Clone SK3; PE‐CyTM7), and CD279 (PD‐1) (Clone MIH4; PE) cells were purchased from BD Biosciences. Samples were run in BD FACS Canto II and the data were analyzed using the FACS Diva software for PD‐1 expression on CD4^+^ T cells (percentage and mean fluorescence intensity [MFI]). Lymphocytes were first gated out through the forward and side scatter and then CD3^+^ CD4^+^ T lymphocytes were gated out for PD‐1 expression analysis. The threshold of PD‐1 percentage and MFI in analyzing the relationship between PD‐1 expression and prognosis was based on the ROC curve, in which a highly specific cutpoint in the ROC curve was used as the threshold.

### Real‐time PCR assay

Total RNAs were extracted from mononuclear cells and were reverse transcribed into cDNAs. Primers were synthesized by the Shanghai Sangon Company. The nucleotide sequences of the primers were as follows: ZAP70 primers (forward primer: 5′‐ CGAGCGTGTATGAGAGCCC, reverse primer: 5′‐ ATGAGGAGGTTATCGCGCTTC); PI3K primers (forward primer: 5′‐ CCACGACCATCATCAGGTGAA, reverse primer: 5′‐ CCTCACGGAGGCATTCTAAAGT); NFAT primers (forward primer: 5′‐ CACCGCATCACAGGGAAGAC, reverse primer: 5′‐ GCACAGTCAATGACGGCTC); GAPDH primer (forward primer: 5′‐ GGAGCGAGATCCCTCCAAAAT, reverse primer: 5′‐ GGCTGTTGTCATACTTCTCATGG). Quantitative RT‐PCR was performed using the SYBR Green PCR Master Mix on Roche lightcycler 480. The reaction conditions for real‐time PCR were as follows: 95°C for 5 min, one cycle; 95°C for 10 s, 60°C for 15 s, 72°C for 15 s, 45 cycles; 95°C for 5 s, 65°C for 1 min, and the fluorescence signal was collected at 97°C; 40°C for 10 s. Samples were measured independently and the individual values were averaged. Data analysis was based on the 2^−ΔΔCt^ method.

### Immunohistochemistry

Paraffin sections of 31 patients were ~5 μm thick. PD‐1 immunohistochemical staining (NAT, 1:50; Abcam, Cambridge, MA) was completed using an automatic immunohistochemistry stainer (Ventana, Tucson, AZ) with high‐pressure antigen retrieval method. All stained sections were independently scored by the two pathologists who were not given patient clinical or prognostic information. For each sample, five representative optical fields (highest PD‐1 staining) were selected for analysis. PD‐1 was located on the cell membrane and the result was calculated according to the percentage of PD‐1‐positive cells (negative: PD‐1‐positive cells <1%, 0 points; weak positive: 1% <PD‐1‐positive cells <25%, 1 point; positive: 26% <PD‐1‐positive cells <49%, 2 points; strongly positive: PD‐1 positive cells >50%, three points) and PD‐1 staining intensity (uncolored, 0 points; light colored, 1 point; medium colored, 2 points; strongly colored, 3 points). The H score was calculated as PD‐1 staining intensity × percentage of positive cells and all patients were divided into categories as negative (H score: 0), weakly positive (H score: 1–4), or strongly positive (H score: ≥5), similar to methods previously reported 10.

### Follow‐up

Follow‐up methods included both telephone‐based follow‐ups and hematology clinic follow‐ups. All patients were monitored until September 20, 2015, with 3–14 months of follow‐up time and a median follow‐up time of 7.5 months. Event‐free survival (EFS) refers to the time from disease diagnosis to the occurrence of any events, including death, disease progression, and the occurrence of fatal side effects. Overall survival (OS) is the time from diagnosis to all‐cause death.

### Statistical methods

SPSS 16.0 software (Chicago, USA) and Graphpad Prism 5 (San Diego, USA) were used for statistical analysis. Qualitative data were presented as numbers and percentages. Measured data were presented as median (interquartile range), and analyzed by the nonparametric Mann–Whitney *U* test. Survival curves were plotted using the Kaplan–Meier method. *P* values <0.05 were considered to be statistically significant, and *P* values ≥0.05 were considered not significant (NS).

## Results

### Clinical data

Sixty cases of newly diagnosed DLBCL patients were within a median age of 60 years (24–81 years) at a male: female ratio of 9:11. According to the international prognostic index (IPI) score, 26.67% of patients were at low risk (IPI ≤ 1), while medium and high‐risk patients accounted for 73.33% of all cases. Based on the subtype of lymphoma, 46.67% of patients were GCB type, 41.67% were ABC type, and 11.67% were not categorized of all cases (Table 1). Thirty‐nine cases of healthy controls were within a median age of 40 years (12–61 years) at a male: female ratio of 6:7.

**Table 1:** Clinical parameters of 60 cases of patients newly diagnosed with diffuse large B‐cell lymphoma

Clinical parameters	Case	Percentage (%)
Age		
≤60	31	51.67
>60	29	48.33
Gender		
Male	27	45
Female	33	55
Diffuse large B‐cell lymphoma symptom		
Negative	28	46.67
Positive	32	53.33
International prognostic index		
Low risk (0–1 point)	16	26.67
Medium/high risk (2–5 point)	44	73.33
Type		
GCB	28	46.67
ABC	25	41.67
Stage		
I–II	16	26.67
III–IV	44	73.33

### PD‐1 expression levels on the surface of peripheral blood CD4^+^ T lymphocytes of DLBCL patients

**Figure 1:**
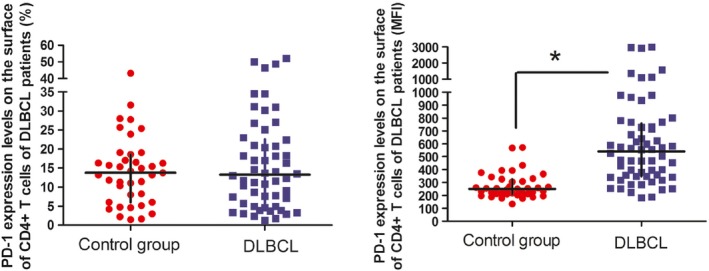
PD‐1 expression levels on the surface of CD4^+^ T cells of diffuse large B‐cell lymphoma patients. *P < 0.05, compared to the control group.

MFI of PD‐1 on the surface of CD4^+^ T cells of DLBCL patients was 541.5 (range: 348.25–758.75), significantly higher than 250 (range: 211–326) in the healthy controls (*P* < 0.001; Fig. 1).

**Figure 2:**
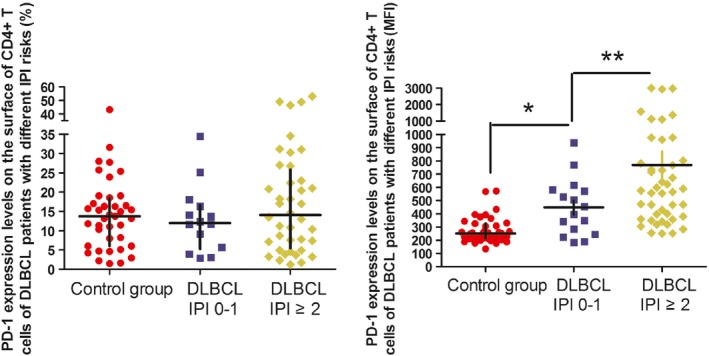
PD‐1 expression levels on the surface of CD4^+^ T cells of diffuse large B‐cell lymphoma patients with different international prognostic index risk. *P < 0.05, compared to the control group. **P < 0.05, compared to the low‐risk group.

**Figure 3:**
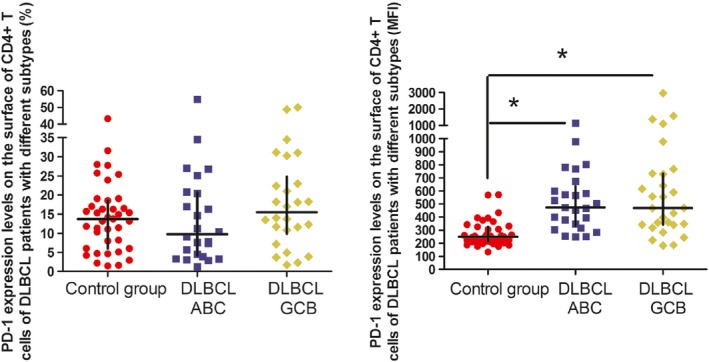
PD‐1 expression levels on the surface of CD4^+^ T cells of diffuse large B‐cell lymphoma patients with different subtypes. *P < 0.05, compared to the control group.

MFI of PD‐1 on the surface of CD4^+^ T cells in low‐risk and medium/high‐risk DLBCL patients were 425 (range: 254–579.25) and 570 (range 365.5–796.75), respectively, significantly higher than 250 (range: 211–326) in healthy controls (*P* = 0.003 and *P* < 0.001, respectively). Similarly, MFI of PD‐1 on the surface of CD4^+^ T cells in ABC‐ and GCB‐type DLBCL patients were 473 (range: 333.5–635) and 468.5 (range: 341.25–732.75), respectively, which was also significantly higher than the healthy controls (*P* < 0.001 and *P* < 0.001, respectively). Interestingly, there were significant differences in MFI of PD‐1 expression between the low‐risk and medium/high‐risk DLBCL patients (*P* = 0.029). However, the differences were not statistically significant in PD‐1 expression between the ABC‐ and GCB‐type DLBCL patients (Figs. 2 and 3).

### ZAP70, PI3K, and NFAT mRNA expression levels in peripheral blood mononuclear cells from DLBCL patients

**Figure 4:**
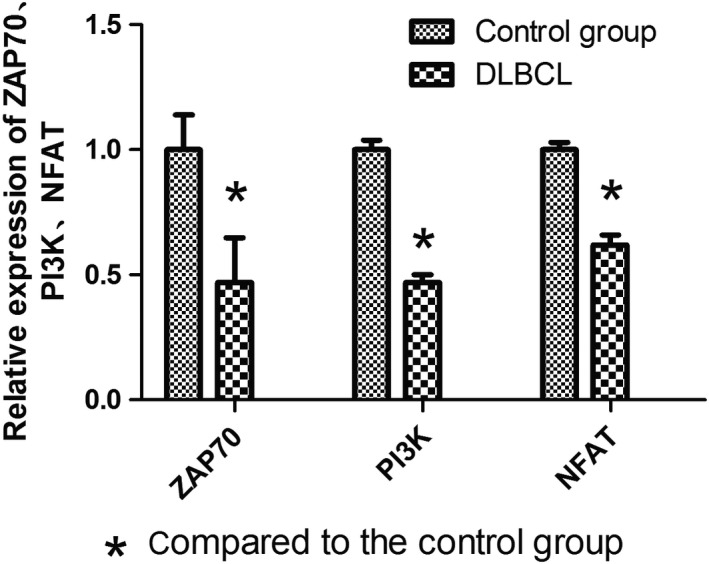
The mRNA expression levels of key downstream molecules of T‐cell receptors in diffuse large B‐cell lymphoma patients.

**Figure 5:**
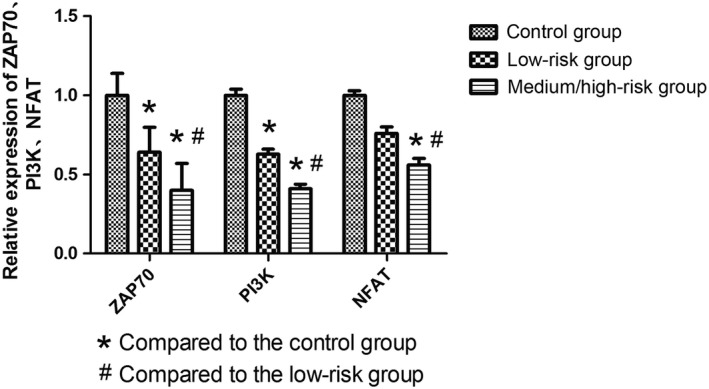
The mRNA expression levels of key downstream molecules of T‐cell receptors in diffuse large B‐cell lymphoma patients with international prognostic index scores.

The ZAP70, PI3K, and NFAT mRNA expression levels in peripheral blood mononuclear cells from the DLBCL patients (45 cases) were 0.47, 0.47, and 0.62 times, respectively, of the expression in the healthy controls (11 cases; *P* < 0.05) (Fig. 4). In the low‐risk DLBCL patients (15 cases), the ZAP70, PI3K, and NFAT mRNA expression levels were 0.64, 0.63, and 0.76 times, respectively, of the healthy controls (*P* < 0.05, *P* < 0.05, and *P* = 0.118, respectively). Additionally, the ZAP70, PI3K, and NFAT mRNA expression levels in the medium/high‐risk DLBCL patients (30 cases) were 0.397, 0.41, and 0.56 times, respectively, of the healthy controls (*P* < 0.05). In comparing the patients, there was a significant reduction in ZAP70, PI3K, and NFAT expression in the medium/high‐risk patients compared to the low‐risk patients (*P* < 0.05; Fig. 5).

In the ABC‐type DLBCL patients (19 cases), the ZAP70, PI3K, and NFAT mRNA expression levels were 0.43, 0.48, and 0.57 times, respectively, of the amount in the control group (*P* < 0.05), while the expression levels in the GCB‐type patients (21 cases) were 0.49, 0.46, and 0.65 times, respectively, of the control group (*P* < 0.05). There was no significant difference between the two subtypes in the mRNA expression levels (*P* = NS).

### Comparison of PD‐1 expression between CD4^+^ T lymphocytes and tumor microenvironment biopsies from individual patients

Eleven patients with the PD‐1 expression in tumor microenvironment were negative, whereas the median percentage of PD‐1 expression on the surface of peripheral blood CD4^+^ T cells was 13.5% (range: 3.9–34.55%) and the median MFI was 640 (range: 182–1377). Fourteen patients with the PD‐1 expression were weakly positive in tumor microenvironment, meanwhile the median percentage and MFI of PD‐1 on CD4^+^ T cells were 17.28% (range: 3.25–46.45%) and 398.5 (range: 244–1590). Six patients were strongly positive, and the median percentage and MFI of PD‐1 on CD4^+^ T cells were 9% (range: 3.7–50%) and 560 (range: 348–1580). Therefore, PD‐1 expression on the surface of peripheral blood CD4^+^ T cells is not consistent with the expression observed from tumor microenvironments in individual patients. In tumor microenvironments, PD‐1 expression was not only restricted to CD4^+^ T cells but was also observed from a variety of other immune cells, including macrophages, natural killer (NK) cells, and CD8^+^ T cells.

### PD‐1 expression on the surface of CD4^+^ T lymphocytes and the prognosis of DLBCL patients

**Figure 6:**
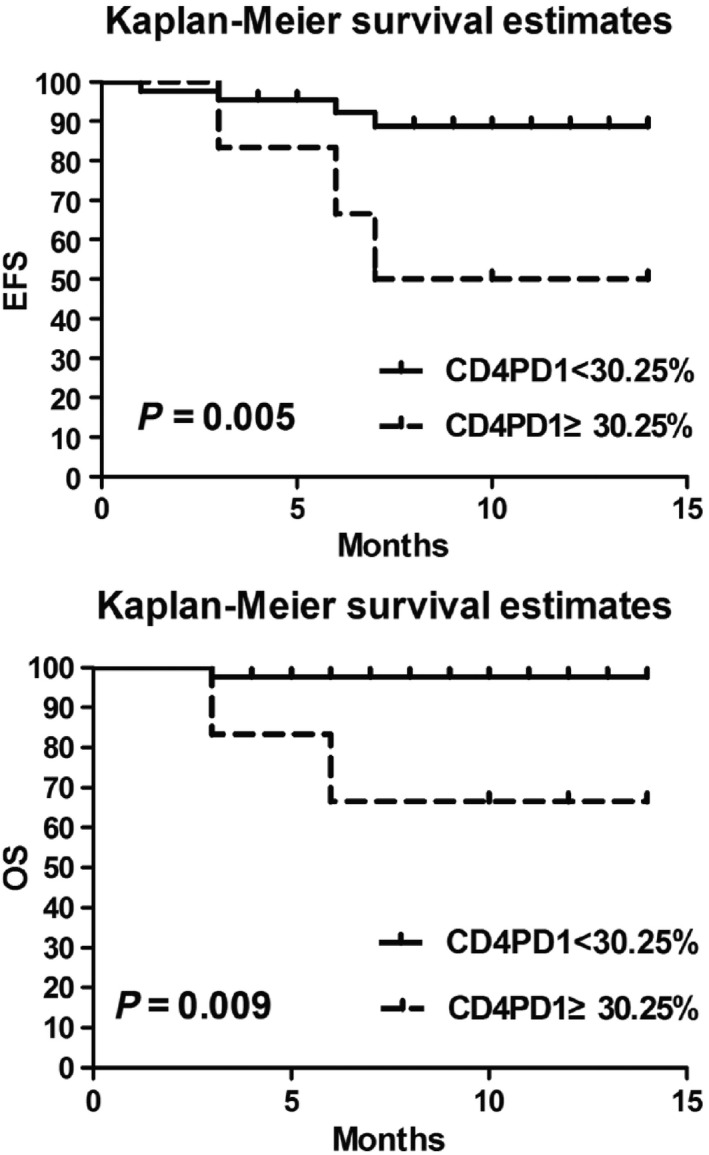
Comparison between PD‐1 percentages of CD4^+^ T cells and the survival of diffuse large B‐cell lymphoma patients.

DLBCL patients with the percentage of PD‐1‐positive CD4^+^ T cells ≥30.25% (*n* = 10) demonstrated significantly lower EFS and OS than patients with PD‐1^+^ CD4^+^ T cells <30.25% (*n* = 50; *P* < 0.05) (Fig. 6). The MFI of PD‐1 on CD4^+^ T cells then were analyzed for correlation with prognosis, and patients with PD‐1 MFI ≥977 (*n* = 9) had significantly lower EFS and OS than patients with PD‐1MFI <977, although this difference was not statistically significant (*P* = NS).

### PD‐1 expression within the tumor microenvironment and the prognosis of DLBCL patients

Based on PD‐1 staining in the tumor microenvironments, the patients were divided into positive and negative groups. The EFS and OS were significantly lower in PD‐1‐positive patients in comparison with the negative group, but the difference was not statistically significant (*P* = 0.49, and *P* = 0.46, respectively).

## Discussion

PD‐1/PD‐L1 signaling pathway blocks T lymphocytes activation and plays an important role in tumor immune evasion 11. Recent studies have indicated that anti‐PD‐1 antibody therapy were effective for patients with refractory and relapsed Hodgkin's lymphoma, advanced multiple myeloma, and melanoma 12, 13, 14. Previous studies focusing on the PD ligand‐1 (PD‐L1) in DLBCL patients have revealed that DLBCL patients showed significantly higher plasma‐soluble PD‐L1 than in healthy volunteers 15, 16. Kiyasu J found that the expression of PD‐L1^+^ in malignant cells within the tumor area in DLBCL patients had inferior overall survival compared with PD‐L1^−^ DLBCL 17. However, PD‐1 expression in DLBCL patients and its association with the prognosis are still unclear. This study measured the PD‐1 expression on the surface of peripheral blood CD4^+^ T cells from DLBCL patients and found that DLBCL patients had significantly higher PD‐1 MFI than the normal controls. In addition, compared to low‐risk DLBCL patients, medium/high‐risk DLBCL patients showed significantly higher MFI of PD‐1 on the surface of CD4^+^ T cells. Ramsay et al. 6 have found that, in CLL patients, expression of PD‐1 (% and MFI) on the surface of CD3^+^ T cell increased significantly, and lenalidomide inhibited PD‐L1/PD‐1‐mediated T‐cell dysfunction in vivo, reversing T‐cell inactivation, and thus enhancing immune function in patients. This suggests that PD‐1 high expression on CD4^+^ T cells in DLBCL patients may undermine T‐cell function, thereby leading to poor prognoses.

To further confirm whether T‐cell function in patients was affected, mRNA expression levels of ZAP70, PI3K, and NFAT were measured from peripheral blood mononuclear cells. This study showed that, in comparison with the control group, DLBCL patients had lower mRNA expression of ZAP70, PI3K, and NFAT, while DLBCL medium/high‐risk group demonstrated a stronger reduction in expression than the DLBCL low‐risk patients. Xia Y also suggested that PD‐1 might suppress the expression of ZAP70, PI3K, and NFAT, which subsequently reduces T‐cell function 18, which is consistent with the results from this work.

MacFarlane et al. observed that patients with renal cell carcinoma had significantly increased PD‐1 expression in peripheral blood mononuclear cell subsets and that expression levels in effector T cells correlated with the disease stage. This suggests that PD‐1 expression of peripheral blood mononuclear cells can be used as a biomarker for disease severity and prognosis 19. Based on this rationale, DLBCL patients with PD‐1 expression on the surface of CD4^+^ T cells over 30.25% and over 977 MFI were found to have reduced EFS and OS. Meanwhile, the observed increase in PD‐1 percentage on CD4^+^ T cells surface was linked to poor prognoses.

The correlation between PD‐1 expression on CD4^+^ T cells of DLBCL patients and PD‐1 expression in tumor microenvironment is unclear. PD‐1 expression levels were measured from patient tumor microenvironment by immunohistochemistry and the results indicated that PD‐1 expression on peripheral CD4^+^ cells does not reflect PD‐1 expression on cells in the tumor microenvironment. The interpretation is complicated due to microenvironment which is a complex of different PD‐1^+^ cells that are indistinguishable by normal immunohistochemistry.

The relationship between PD‐1 expression in tumor microenvironment and prognosis was further explored and patients with positive PD‐1 expression within the tumor microenvironment had a reduced EFS and OS compared to the negative patients, however, the difference was not statistically significant. A previous study has shown that PD‐1 expression levels in tumor microenvironment of patients with follicular lymphoma were correlated with patient prognosis 20, 21, 22, but the finding was still controversial. Interestingly, Carreras found that patients with higher PD‐1‐positive cells had a higher 5‐year overall survival and progression‐free survival 20, while two other studies suggested that PD‐1 positivity in T cells of follicular lymphoma was an unfavorable prediction factor for transformation and was associated with poor prognosis 21, 22. Yang found that low PD‐1 expression on the surface of CD4^+^ or CD8^+^ T cells within follicular lymphoma was associated with poor prognosis, but high PD‐1 expression had no association with prognosis 9. Muenst et al. 23 demonstrated that, in classical Hodgkin lymphoma patients, PD‐1 expression on lymphocyte predominant pathologic tissues was positive, and there was significant difference in prognosis between PD‐1‐positive cells >23/mm^2^ (29 deaths/75 patients) and PD‐1‐positive cells <23/mm^2^ (9 deaths/23 patients) (*P* = 0.005). In view of above, it is seemed that the relationship between PD‐1 expression in tumor environment and prognosis are complex. The underlying reason may be that PD‐1 is expressed on different cells (T cells, NK cells, macrophages) in tumor microenvironment, all of these types of immune cells offer different contributions to the anticancer activity. It is valuable for us to explore the relationship between the PD‐1 expression on different cells in tumor microenvironment and prognosis in the future study.

In summary, this study indicates that DLBCL patients with high PD‐1 expression on CD4^+^ T cells might damage T‐cell function, thereby leading to poor prognosis, while PD‐1 expression on peripheral blood CD4^+^ T cells does not reflect PD‐1 expression on cells in the tumor microenvironment. This study was limited by the fact that only mRNA levels of three key elements (ZAP70, PI3K, and NFAT) were measured and that protein expression was not included in the study. Moreover, patients were followed up for a short time and the longest follow‐up time was 14 months. Therefore, future studies will extend the follow‐up time and use a variety of methods to assess the T‐cell function of patients. Meanwhile, We can also further explore the PD‐1 expression on specific cell types within the tumor microenvironment, and it is significant to examine the relationship between PD‐1 expression on specific cell types and prognosis.

## Conflict of Interest

None.

## References

[1] Sun, J. , Q. Yang , Z. Lu , M. He , L. Gao , M. Zhu , et al. 2012 Distribution of lymphoid neoplasms in China: analysis of 4638 cases according to the World Health Organization classification. Am. J. Clin. Pathol. 138:429–434. 22912361 10.1309/AJCP7YLTQPUSDQ5C

[2] Raut, L. S. , and P. P. Chakrabarti . 2014 Management of relapsed‐refractory diffuse large B cell lymphoma. South Asian J. Cancer 3:66–70. 24665451 10.4103/2278-330X.126531 PMC3961873

[3] Gotsman, I. , A. H. Sharpe , and A. H. Lichtman . 2008 T‐cell costimulation and coinhibition in atherosclerosis. Circ. Res. 103:1220–1231. 19028921 10.1161/CIRCRESAHA.108.182428 PMC2662382

[4] Parry, R. V. , J. M. Chemnitz , K. A. Frauwirth , A. R. Lanfranco , I. Braunstein , S. V. Kobayashi , et al. 2005 CTLA‐4 and PD‐1 receptors inhibit T‐cell activation by distinct mechanisms. Mol. Cell. Biol. 25:9543–9553. 16227604 10.1128/MCB.25.21.9543-9553.2005 PMC1265804

[5] Oki, Y. , D. Buglio , J. Zhang , Y. Yang , S. Zhou , A. Sureda , et al. 2014 Immune regulatory effects of panobinostat in patients with Hodgkin lymphoma through modulation of serum cytokine levels and T‐cell PD1 expression. Blood Cancer J. 4:e236. 25105535 10.1038/bcj.2014.58 PMC4219471

[6] Ramsay, A. G. , A. J. Clear , R. Fatah , and J. G. Gribben . 2012 Multiple inhibitory ligands induce impaired T‐cell immunologic synapse function in chronic lymphocytic leukemia that can be blocked with lenalidomide: establishing a reversible immune evasion mechanism in human cancer. Blood 120:1412–1421. 22547582 10.1182/blood-2012-02-411678 PMC3423779

[7] Rosenblatt, J. , B. Glotzbecker , H. Mills , B. Vasir , D. Tzachanis , J. D. Levine , et al. 2011 PD‐1 blockade by CT‐011, anti‐PD‐1 antibody, enhances ex vivo T‐cell responses to autologous dendritic cell/myeloma fusion vaccine. J. Immunother. 34:409–418. 21577144 10.1097/CJI.0b013e31821ca6ce PMC3142955

[8] Armand, P. , A. Nagler , E. A. Weller , S. M. Devine , D. E. Avigan , Y. B. Chen , et al. 2013 Disabling immune tolerance by programmed death‐1 blockade with pidilizumab after autologous hematopoietic stem‐cell transplantation for diffuse large B‐cell lymphoma: results of an international phase II trial. J. Clin. Oncol. 31:4199–4206. 24127452 10.1200/JCO.2012.48.3685 PMC4878008

[9] Yang, Z. Z. , D. M. Grote , S. C. Ziesmer , B. Xiu , A. J. Novak , S. M. Ansell , et al. 2015 PD‐1 expression defines two distinct t cell subpopulations in follicular lymphoma that differentially impact patient survival. Blood Cancer J. 5:e281. 25700246 10.1038/bcj.2015.1 PMC4349259

[10] Berghoff, A. S. , G. Ricken , G. Widhalm , O. Rajky , K. Dieckmann , P. Birner , et al. 2015 Tumour‐infiltrating lymphocytes and expression of programmed death ligand 1 (PD‐L1) in melanoma brain metastases. Histopathology 66:289–299. 25314639 10.1111/his.12537

[11] Pedoeem, A. , I. Azoulay‐Alfaguter , M. Strazza , G. J. Silverman , and A. Mor . 2014 Programmed death‐1 pathway in cancer and autoimmunity. Clin. Immunol. 153:145–152. 24780173 10.1016/j.clim.2014.04.010

[12] Ansell, S. M. , A. M. Lesokhin , I. Borrello , A. Halwani , E. C. Scott , M. Gutierrez , et al. 2015 PD‐1 blockade with nivolumab in relapsed or refractory Hodgkin's lymphoma. N. Engl. J. Med. 372:311–319. 25482239 10.1056/NEJMoa1411087 PMC4348009

[13] Wolchok, J. D. , H. Kluger , M. K. Callahan , M. A. Postow , N. A. Rizvi , A. M. Lesokhin , et al. 2013 Nivolumab plus ipilimumab in advanced melanoma. N. Engl. J. Med. 369:122–133. 23724867 10.1056/NEJMoa1302369 PMC5698004

[14] Hamid, O. , C. Robert , A. Daud , F. S. Hodi , W. J. Hwu , R. Kefford , et al. 2013 Safety and tumor responses with lambrolizumab (anti‐PD‐1) in melanoma. N. Engl. J. Med. 369:134–144. 23724846 10.1056/NEJMoa1305133 PMC4126516

[15] Chen, Y. , Q. Wang , B. Shi , P. Xu , Z. Hu , L. Bai , et al. 2011 Development of a sandwich ELISA for evaluating soluble PD‐L1(CD274) in human sera of different ages as well as supernatants of PD‐L1 + cell lines. Cytokine 56:231–238. 21733718 10.1016/j.cyto.2011.06.004

[16] Rossille, D. , M. Gressier , D. Damotte , D. Maucort‐Boulch , C. Pangault , G. Semana , et al. 2014 High level of soluble programmed cell death ligand 1 in blood impacts overall survival in aggressive diffuse large B‐cell lymphoma: results from a French multicenter clinical trial. Leukemia 28:2367–2375. 24732592 10.1038/leu.2014.137

[17] Kiyasu, J. , H. Miyoshi , A. Hirata , F. Arakawa , A. Ichikawa , D. Niino , et al. 2015 Expression of programmed cell death ligand 1 is associated with poor overall survival in patients with diffuse large B‐cell lymphoma. Blood 126:2193–2201. 26239088 10.1182/blood-2015-02-629600 PMC4635115

[18] Xia, Y. , L. Jeffrey Medeiros , and K. H. Young . 2016 Signaling pathway and dysregulation of PD1 and its ligands in lymphoid malignancies. Biochim. Biophys. Acta 1865:58–71. 26432723 10.1016/j.bbcan.2015.09.002 PMC4733614

[19] MacFarlane, A. W. IV , M. Jillab , E. R. Plimack , G. R. Hudes , R. G. Uzzo , S. Litwin , et al. 2014 PD‐1 expression on peripheral blood cells increases with stage in renal cell carcinoma patients and is rapidly reduced after surgical tumor resection. Cancer Immunol. Res. 2:320–331. 24764579 10.1158/2326-6066.CIR-13-0133 PMC4007343

[20] Carreras, J. , A. Lopez‐Guillermo , G. Roncador , N. Villamor , L. Colomo , A. Martinez , et al. 2009 High numbers of tumor‐infiltrating programmed cell death 1‐positive regulatory lymphocytes are associated with improved overall survival in follicular lymphoma. J. Clin. Oncol. 27:1470–1476. 19224853 10.1200/JCO.2008.18.0513

[21] Richendollar, B. G. , B. Pohlman , P. Elson , and E. D. Hsi . 2011 Follicular programmed death 1‐positive lymphocytes in the tumor microenvironment are an independent prognostic factor in follicular lymphoma. Hum. Pathol. 42:552–557. 21237493 10.1016/j.humpath.2010.08.015

[22] Smeltzer, J. P. , J. M. Jones , S. C. Ziesmer , D. M. Grote , B. Xiu , K. M. Ristow , et al. 2014 Pattern of CD14 + follicular dendritic cells and PD1 + T cells independently predicts time to transformation in follicular lymphoma. Clin. Cancer Res. 20:2862–2872. 24727328 10.1158/1078-0432.CCR-13-2367 PMC4058762

[23] Muenst, S. , S. Hoeller , S. Dirnhofer , and A. Tzankov . 2009 Increased programmed death‐1 + tumor infiltrating lymphocytes in classical Hodgkin lymphoma substantiate reduced overall survival. Hum. Pathol. 40:1715–1722. 19695683 10.1016/j.humpath.2009.03.025

